# Can we hit prenatal depression and anxiety through HIIT? The effectiveness of online high intensity interval training in pregnant women during the COVID-19 pandemic: a randomized controlled trial

**DOI:** 10.1186/s13102-022-00610-2

**Published:** 2022-12-22

**Authors:** Dominika Wilczyńska, Tamara Walczak-Kozłowska, Łukasz Radzimiński, Miguel Ángel Oviedo-Caro, Rita Santos-Rocha, Anna Szumilewicz

**Affiliations:** 1 Department of Physical Culture, Gdansk University of Physical Education and Sport, Gdansk, Poland; 2 Department of Neuropsychology, Institute of Psychology, University of Gdansk, Gdansk, Poland; 3 Department of Physical Education and Sport, University of Seville, Seville, Spain; 4 Sport Sciences School of Rio Maior (ESDRM), Polytechnic Institute of Santarém, Rio Maior, Portugal; 5 Interdisciplinary Centre for the Study of Human Performance (CIPER), Faculty of Human Kinetics (FMH), University of Lisbon, lisboa, Portugal

**Keywords:** Pregnancy, Exercise, High intensity interval training, Emotional condition, Coronavirus

## Abstract

**Background:**

In recent years high intensity interval training (HIIT) has grown in popularity. However, it rarely represents training interventions in experimental studies in pregnant populations. Therefore, in this study we aimed to assess changes in depressive symptoms, fear of childbirth, fear of Covid-19 and quality of life after an 8-week supervised online HIIT program, compared to an educational (self-performed physical activity) program.

**Methods:**

We conducted a randomized control trial among 54 Caucasian women in uncomplicated, singleton pregnancy (age 32 ± 4 years, 22 ± 4 week of gestation; mean ± SD). There were 34 women in the experimental group, who participated in an 8-week high intensity interval training program (HIIT group). The comparative group was constituted of 20 pregnant women who attended 8-week educational program (EDU group).

**Results:**

The most important finding was that mental health improved somewhat in both groups after the intervention, but only the HIIT group improved statistically significantly. The positive trends in lowering the severity of depressive symptoms, fear of childbirth, and fear of Covid-19 were observed in both groups. However, the positive response to the intervention was stronger in the EDU group. As a secondary outcome, there was a significant decrease in cardiorespiratory fitness level in the EDU group, while the HIIT group maintained unchanged level of maximal oxygen uptake.

**Conclusions:**

HIIT seems to be beneficial for women with uncomplicated pregnancies to maintain adequate quality of life and mental health. However, more research is needed to determine the effectiveness of prenatal HIIT in pregnant women in various psychological conditions.

*Trial registration*: We conducted this study in Poland, in 2021. It was approved by the Bioethics Commission at the District Medical Chamber in Gdansk (KB-8/21). The full study protocol was registered in ClinicalTrials.gov (NCT05009433).

## Introduction

Pregnancy is an important phase in a woman's existence, and a woman's physical and mental health status affects both her and her child's life. During this period several anatomical, hormonal, psychological, and lifestyle and social role changes appear and may potentially affect women’s daily life. Related to these changes, there is an increased risks of depressive disorders during pregnancy, which applies to as many as 23% of pregnant women [1]. Another common problem is fear of childbirth, which complicates about 20% of pregnancies [2]. Moreover, the aforementioned changes and the pregnancy-related symptoms [3] have potential consequences on women’s health-related quality of life. That makes their perception of quality of life lower compared with nonpregnant women of similar age, especially in the physical function [4]. According to some authors, the physical component of quality of life decreases throughout pregnancy while the mental component remains stable [5].

Research over the past 30 years has shown that regular physical activity during pregnancy has a positive effect on the physical and psychological condition of the pregnant woman, on pregnancy and on fetal development [6–10]. Among mental benefits are reduced stress, anxiety, and depression as well as well-being improvement. Despite the aforementioned benefits, women have the tendency to reduce their physical activity during the course of pregnancy, which is often associated with the intensification of depressive symptoms [11]. Maternal depression can lead to premature birth, increased stress hormones in infants, and may cause difficulties in establishing a bond with the baby (higher risk of developing insecure attachment style in an infant); negatively affecting its physical and mental development [1, 12]. Based on a meta-analysis of 131,406 pregnant women, Davenport et al. [7] showed that physical activity during pregnancy reduces the risk of perinatal depression by as much as 67%. This result corresponds with the works of other authors [13, 14]. Guszkowska et al. [15] demonstrated that women who participated in an exercise program during pregnancy significantly reduced their fear of childbirth compared to those who attended traditional childbirth classes. Women fear childbirth mainly because they are afraid of pain [15]. Exercising can help reduce fear of childbirth by improving the ability to control breathing and muscle tension and decreasing the level of anxiety, both as a trait and as a state [16]. In addition, physical activity is associated with better quality of life perception during pregnancy [17, 18].

In the COVID-19 pandemic, the mental condition of many future mothers was visibly weaker [19]. This results, among others, in the decline of physical activity levels [20]. The restrictions of the COVID-19 pandemic imposed to reduce infection rates, caused pregnant women to experience disruption not just to their daily lives but also their pregnancy healthcare experience, and led to social confinement. As Atkinson et al. [21] underlined in their work, this also meant substantial changes to how, when, and why pregnant women were involved and practiced physical activity and exercise. Moreover, many pregnant women fear COVID‐19. Therefore, they worry about visiting labor and delivery wards and even delay or cancel antenatal visits and face-to-face consultations during the pandemic [22–25]. The anxiety increases due to the thought that the virus would be transmitted to their fetus by vertical transmission [26]. Therefore, it is necessary to determine different activities and ways of support for pregnant women to deal with emotions and stress caused by these extraordinary circumstances. Lebel et al. [27] reported in the studies in 1.987 pregnant women in Canada that regularly practiced physical activity and social support lowered the likelihood of symptoms of anxiety and depression during the pandemic. It seems crucial to popularize pro-health exercises in pregnant women from various social groups, especially those with a low degree of involvement in physical activity before the pandemic. Women should be encouraged to continue exercising until the day of delivery. Physical activity is recommended during pregnancy owing to the significant health benefits for mothers and their offspring [21, 28].

While it is well known that physical activity and exercise have health benefits for pregnant women, simultaneously, a question arises which kind of training is most suitable for future mothers? According to current guidelines published by credible obstetrics, gynecology, and sports medicine institutions, including the World Health Organization, women should perform at least 150 min of moderate intensity aerobic physical activity throughout the week [29–32]. For moderate-intensity exercise, ratings of perceived exertion should be 13–14 (somewhat hard) on the Borg ratings of perceived exertion scale [33]. According to some researchers, aerobic exercises such as walking, especially outdoors, are most beneficial for revitalizing feelings of well-being of pregnant women [7, 34]. Rodrigues-Ayllon et al. [35] demonstrated that the moderate-to-vigorous physical activity was negatively associated with depression in pregnant women. However, little is known so far about the effects of high-intensity exercise on psychosocial condition of mothers-to-be [35].

In recent years, high intensity interval training (HIIT) has grown in popularity [36], inter alia due to its health benefits. There is a variety of HIIT protocols: they are based on short work intervals (< 60 s–8 min) of vigorous (70–90% maximal heart rate or 14–16 of the 6–20 Borg’s rate of perceived exertion scale—RPE) to high intensity (≥ 90% maximal heart rate or ≥ 17 of the 6–20 RPE) interspersed with active (40–70% maximal heart rate or 8–13 of the 6–20 RPE) or passive (cessation of movement) recovery periods (of 1–5 min) [37]. Some authors observed that HIIT used as an acute intervention improved well-being and reduced distress and state of anxiety in patients with depression and schizophrenia [38]. Other researchers found that the HIIT intervention seemed to be more beneficial to reduce depression and anxiety than a moderate intensity training [39, 40]. Contrary to these results, in another study, although HIIT decreased depressive symptoms, it also increased perceived stress [41]. Therefore, the authors suggested that moderate intensity exercise may be an optimal intensity of exercise for the promotion of mental health. Based on a literature review, Kleinert and Bassek [42] concluded that the positive effects of HIIT on the quality of life, depression, anxiety and fatigue are inconsistent. However, they did not find proof of the negative psychological effects of HIIT [42].

Until now, HIIT programs rarely represent training interventions in experimental studies in pregnant populations [43], although the upper limit for the intensity of physical effort for the perinatal period has not been set so far [8, 29, 44]. All the evidence shows that pregnant women can benefit from HIIT programs in the same way, as other populations [43]. Therefore, in this study we decided to investigate the effectiveness of HIIT intervention on selected psychological characteristics among pregnant women. Firstly, we aimed assess the changes in depressive symptoms, fear of childbirth, coronavirus anxiety, and perception of quality of life after an 8-week supervised online HIIT program compared to an educational and self-performed physical activity program. Secondly, we aimed to determine the predictors of these changes. As a secondary outcome we measured the levels of maximal oxygen uptake (VO_2_max) before and after the experiment. Thanks to this, we were able to supplement the psychological parameters analysis with biological indicators, obtaining a more complete insight on the effectiveness of our interventions. The VO_2_max parameter reflects the physical work capacity of the future mother and her ability to provide the fetus with oxygen. It may significantly affect the development of the pregnancy and the baby [45].

## Methods

The present study was a randomized control trial among 54 Caucasian female participants in uncomplicated, singleton pregnancy (age 32 ± 4 years, 22 ± 4 week of gestation; mean ± SD) who responded to our mass media invitation and volunteered for the study. The study was conducted in the Laboratory of Physical Effort and Genetics in Sport in Gdansk, Poland in 2021. There were 34 participants in the experimental group, who participated in an 8-week high-intensity interval training program (HIIT group). The comparative group consisted of 20 pregnant participants who attended 8-week educational program on a healthy lifestyle and physical activity in the perinatal period (EDU group). The eligibility criterion was a course of pregnancy allowing participation in physical activities adapted to pregnant participants, confirmed by the routine obstetric consultation. Exclusion criteria were contraindications to increased physical effort or other conditions that, according to the researchers, could threaten the health or safety of the participants or could significantly affect the quality of the collected data.

Before and after the intervention we collected data from our study participants, using following tools:

### Beck depression inventory-II (BDI-II)

The occurrence and severity of depression symptoms using BDI-II (Beck Depression Inventory-II). The BDI-II is a patient-rated 21-item inventory to evaluate depressive symptoms. For each item, the participants are required to rate on a scale of 4 ranging from 0 to 3 the severity of the symptoms in the last two weeks. Scores can range from 0 to 63. BDI-II classification is as follows: 0–13: no depression; 14–19: mild depression; 20–28: moderate depression; 29–63: severe depression. The BDI-II has established psychometric properties [46]. In the current study, the Cronbach alpha for the HIIT group and EDU group for depressive symptoms was 0.78 and 0.73, respectively.

### Fear of childbirth

The fear of childbirth was measured with the Childbirth Attitudes Questionnaire (CAQ), developed from a questionnaire designed to measure fear of childbirth by Areskog et al. [47]. The CAQ is a 16-item questionnaire, with a 4-point Likert scale. The item scores are summed to provide a total score (range: 16–64) with higher scores indicating higher levels of fear of childbirth [47]. The Cronbach alpha for both HIIT group and EDU group was 0.90.

### 12-Item short form health survey (SF-12)

Health-related quality of life was assessed with 12-item Short Form Health Survey (SF-12) instrument which includes a physical (PCS) and a mental (MCS) scale [48]. The SF-12 is a self-administered questionnaire, which measures health status. Responses to questions are dichotomous (yes/no), ordinal (excellent to poor), or expressed by frequency (always to never). The answers to this 12-item questionnaire allow calculation of Physical Component Summary (PCS) and Mental Component Summary (MCS) scores. In the absence of response to a single question of these subscales, the score cannot be calculated. The higher the score, the better the health status. The SF-12 reliability from the study of Ware et al. [48] is 0.93 Cronbach alpha. In the current study, the Cronbach alpha for HIIT group and EDU group was 0.75 and 0.67, respectively.

### Fear of COVID-19 scale (FCV-19S)

The authors used the Fear of COVID-19 Scale (FCV-19S) of Ahorsu et al. [49]. The scale consists of 7 items, which are ranged on a 5-point Likert scale ranging from 1—strongly disagree to 5—strongly agree. The scale has single factor structure, with internal consistency α = 0.82 [49]. Scores range from 7 to 35, and the higher the score the worse the outcome. In the current study, the Cronbach alpha for HIIT group and EDU group for coronavirus fear was 0.84 and 0.87, respectively.

### International physical activity questionnaire (IPAQ)

The level of physical activity was measured by the short form of International Physical Activity Questionnaire [50]. This questionnaire, which has shown acceptable measurement properties, provides information on weekly PA levels in multiples of the resting metabolic rate (METs). Based on IPAQ outcomes, we categorized the pregnant participants using three levels (categories) of PA: low (inactive participants), moderate (accumulating a minimum recommended level of PA) and high (exceeding the minimum recommended level of PA) [51, 52].

### Progressive maximal exercise test

Maternal oxygen consumption during exercise was measured during a progressive maximal test on a cycloergometer with electronically regulated load (Viasprint 150P) and respiratory gas analyzer (Oxycon Pro, Erich JAEGER GmbH, Germany). We presented the test protocol in our previous study [53]. As maximal oxygen capacity (VO_2_max) we treated the highest value of oxygen uptake, which was maintained for 15 s. The anaerobic threshold (AT) values, such as oxygen uptake at AT (VO2/AT) and heart rate at AT (HR/AT) were established using the V-slope method [54].

### Experimental training and educational interventions

The HIIT intervention consisted of attending three 60-min training sessions a week for eight weeks. The warm-up together with educational tips on how to perform exercises in the main part lasted 7–10 min. The main part (15–20 min) was conducted in the form of high intensity intervals. Based on the progressive maximal exercise test we determined the individual heart rate at an aerobic threshold (HR/AT) for each woman. On average, the HR/AT was set at 87% ± 5 of maximal heart rate. During the exercise sessions the participants used the heart rate monitor watches (Polar RS400, Finland) to observe their individual exercise intensity. They were supposed to exceed the value of HR/AT in workout intervals for as long as they felt comfortable. The exercise intensity was also monitored with the use of the 0–10 Borg Rating of Perceived Exertion (RPE) [33] and the Talk Test [55].

The workout intervals consisted of exercises involving the main muscle groups (e.g., squats, lunges, jumps, combined with the upper body movements). They lasted for 30–60 s, alternating with a 30–60 s rest break, in the ratio of exercise time to rest 1:2, 1:1 or 2:1, according to the individual capabilities of the participant and taking into account the training progression and stage of pregnancy. Following the interval part of the training, participants performed resistance, postural, neuromotor (e.g., body balance) and stretching exercises (5–10 min). The cool down included pelvic floor muscle exercises and preparation-for-birth exercises, e.g., birth position and breathing exercises (5–10 min) and also relaxation and visualization of pregnancy and childbirth (5–15 min). No equipment was used during exercises and only resistance of own body was applied. Participants could attend the exercise program regardless of their level of fitness or exercise capacity, as well as the level of motor skills (based on the diagnostic exercise tests, the exercise program was tailored to the individual exercise capabilities) [56, 57].

Group HIIT sessions were held online from 9.30 to 10.30 a.m. using the MS Teams® platform on Mondays, Wednesdays and Fridays, except one Monday which was a holiday (23 sessions in total). Participants attended 19 ± 4 sessions on average (80% of the entire training program). Preceding their participation, participants were trained on the MS Teams application and on how to exercise safely at home. The HIIT intervention was supplemented by educational class once a week. The sessions were conducted by the principal researcher, who is a graduated fitness professional certified Pregnancy and Postnatal Exercise Specialist according to the European educational standard for this profession [58]. We used email and phone contact to monitor the adherence to the program.

The comparative group (EDU group) was constituted of 20 pregnant participants who attended educational sessions on a healthy lifestyle, physical activity in the perinatal period and selected aspects of pregnancy and motherhood. The educational program was the same as for the HIIT group. Educational classes ware conducted online in real time, once a week for 8 weeks. We encouraged participants from the EDU group to individually undertake exercise and fulfill at least the recommended level of physical activity (minimum 150 min per week of moderate to vigorous intensity). We asked them to keep a diary of all their physical activity (including both structured exercise sessions and daily activities lasting at least 10 min, such as cleaning the house, gardening, shopping). The educational group did not monitor the intensity with heart rate monitors, but used the RPE scale and Talk Test. We recommended exercise intensity at a level in which they felt a marked increase in breathing frequency, but until their breathing interfered with their conversation. On average, the participants reported 19 bouts of physical activity with an average intensity of 6 ± 1 on the 0–10 RPE scale.

During the entire experiment, all study participants remained under standard obstetric care. Both interventions were not associated with any negative effects on the course of pregnancy or on childbirth parameters. Data on obstetric and neonatal outcomes were collected postpartum, using an online questionnaire and based on medical documentation.

We conducted this study in the Laboratory, in 2021. It was performed according to the principles of the WMA Declaration of Helsinki and with the approval of the Bioethics Commission at the District Medical Chamber in Gdansk (KB-8/21). The participants signed the informed consent before testing. The full study protocol was registered in ClinicalTrials.gov (NCT05009433) on 17/08/2021. No important methodological changes were done after trial commencement. In this study we followed standards for transparency, openness, and reproducibility of research [59] and also adhered to the BMC Sports Science, Medicine and Rehabilitation methodological checklist and CONSORT standards [60]. We performed no data manipulations. Materials for this study are available by emailing the corresponding author. The data analysis presented in this work was not preregistered.

**Fig. 1:**
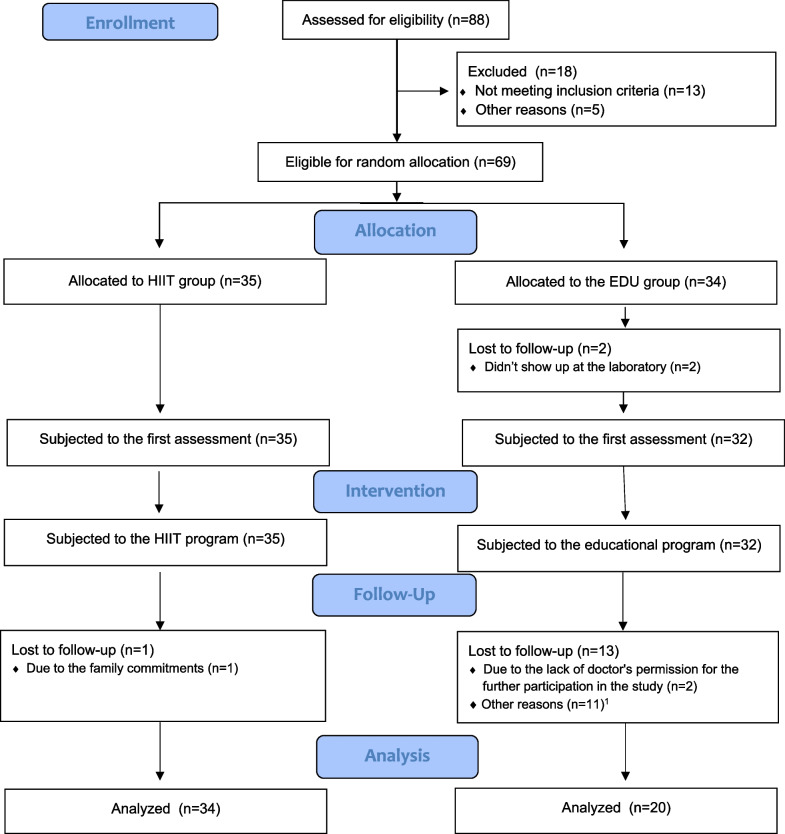
The flow of participants through the study. *Note*. HIIT—high intensity interval training; EDU—educational. ^1^Other reasons: no interest to continue the program (n = 6); preterm birth (n = 1); not feeling well on the day of the second assessment (n = 2); did not provide the reason (n = 2)

The sample size was predetermined by using a power calculation with the software G ∗ power version 3.1.3. The estimated values of the mean and SD from preliminary tests with 9 participants form the HIIT group allowed us to predetermine the minimal sample size of 44 (22 for each group) with an allocation ratio 1:1, a power of 0.8 and alpha of 0.05. The flow of participants through the study is presented in Fig. 1.

Statistical tests were performed using the IBM Statistical Package for the Social Sciences version 26.0 (IBM Corp., Armonk, New York, USA), with the statistical significance set to *p* < 0.050. The analysis of the normality of the distribution of study variables was developed using the Kolmogorov–Smirnov test (K–S test). Inter- and intra-group mean differences were analyzed by the Student’s *t*-test or analysis of variance (ANOVA) test when appropriate. In the case of distributions which were significantly different from the normal distribution, we used the non-parametric Mann–Whitney *U* test and the Wilcoxon *T* test for the assessment of inter- and intra-group differences, respectively. Additionally, Chi-square was used to evaluate the differences in frequencies. We also conducted Intention-to-treat (ITT) analyses using linear interpolation to estimate the results of participants who were lost during the study.

## Results

The characteristics of the participants are gathered in Table 1. The HIIT and EDU groups in terms of age, BMI, physical fitness, and PA levels presented values not statistically different. The EDU group was at slightly higher week of pregnancy. However, we have considered the observed statistically significant difference of 4 weeks between groups of no clinical significance. In the pre-intervention assessment, we did not observe any significant differences between both groups in any of the measured psychosocial parameters (Table 1).

**Table 1:** The characteristics of the study participants

Variable	Group				
	HIIT	EDU	Statistics^1^	*p*-value	Effect size^2^
	n = 34, M ± SD	n = 20, M ± SD			
Age (years)	31 ± 4	32 ± 4	*Z* = − 0.675	0.500	0.185
BMI (height/weight^2^)	24.4 ± 2.8	25.4 ± 3.2	*t* = − 1.141	0.259	0.322
Week of gestation	20 ± 4	24 ± 4	*Z* = − 2.741	**0.006**	0.780
Initial VO_2_max (kg/ml/min)	25.6 ± 4.4	23.7 ± 3.6	*Z* = − 1.505	0.132	0.417
Initial weekly PA (METs)	2625.3 ± 1823.2	2266.0 ± 1816.5	*Z* = − 0.752	0.452	0.206

^1^In case of variables with the distribution close to normal distribution we used parametric testing with Student t test and in case of variables with a distribution significantly different from the normal distribution we used non-parametric testing with Mann–Whitney *U* test

^2^In case of variables with the distribution close to normal distribution we used Cohen’s d for the evaluation of effect sizes and in case of variables with a distribution significantly different from the normal distribution we used rank-biserial correlation

Bold type indicates significant difference in the outcome variable

91% of participants from the HIIT group and 90% from the EDU group had higher educational level. The remaining participants had secondary education. 44% of participants from the HIIT group and 50% from the EDU group had moderate level of physical activity. 38% of participants from the HIIT group and 30% from the EDU group reported high level of physical activity. The remaining 18% of participants from the HIIT group and 20% from the EDU group presented low level of physical activity. Groups did not differ in their educational level (*χ*^2^ = 0.021, *p* = 0.885) and in a category of physical activity measured with the IPAQ (*χ*^2^ = 0.374, *p* = 0.829). At baseline the clinical score for depression symptoms was obtained by 5.9% of participants from the HIIT group and 10% of participants in the EDU group. Whereas during the second assessment the clinical score for depression symptoms was obtained by 5.9% of participants form the HIIT group and none of participants in the EDU group.

### The severity of depressive symptoms

The results obtained in the ANOVA with repeated measures revealed a significant main effect (*F* = 6.530, *p* = 0.014, η^2^ = 0.112, observed power = 0.708), in the absence of an interaction effect (*F* = 0.878, *p* = 0.353, η^2^ = 0.017, observed power = 0.151), in case of the severity of depressive symptoms. However, when controlling for the week of birth (variable entered as a covariate in ANOVA) neither interaction effect (*F* = 1.506, *p* = 0.225, η^2^ = 0.029, observed power = 0.226) nor main effect remained to be significant (*F* = 2.010, *p* = 0.162, η^2^ = 0.038, observed power = 0.285). Further post-hoc analyzes showed that the groups did not differ in the severity of depressive symptoms, both in the first measurement as well as in the second measurement (see Table 2 for the details).

**Table 2:** The severity of depressive symptoms

	Group			95% CI			
	HIIT	EDU	Student’s *t* test	LL	UL	*p*-value	Cohen’s *d*
	n = 34, M ± SD	n = 20, M ± SD					
First measurement	5.68 ± 4.44	5.35 ± 3.69	0.277	− 2.039	2.692	0.783	0.078
Second measurement	4.91 ± 3.49	3.70 ± 2.01	1.620	− 0.289	2.713	0.111	0.399

The change over time was non-significant in both the HIIT group (*t* = 1.620, *p* = 0.115, 95% CI: LL =  − 0.196, UL = 1.725, Cohen’s *d* = 0.278), as well as in the EDU group (*t* = 1.759, *p* = 0.095, 95% CI: LL =  − 0.314, UL = 3.614, Cohen’s *d* = 0.393). ITT analysis confirmed the above outcomes except that there was a significant difference between the first and the second measurement for the EDU group (*t* = 2.545, *p* = 0.016, 95% CI: LL = 0.385, UL = 3.466, Cohen’s *d* = 0.443; *M*_*pre*_ = 5.79, *SD*_*pr*e_ = 4.23; *M*_*post*_ = 3.86, *SD*_*post*_ = 1.73).

Additional analysis with the Chi-square test showed that the groups (HIIT and EDU) did not differ in the presence or absence of clinical symptoms of depression (comparison of the observed and expected frequencies in each category: "occurrence of depressive symptoms", "no depressive symptoms") both in the pre-test (*χ*^2^ = 21.205, *p* = 0.130) and in the post-test (*χ*^2^ = 10.796 *p* = 0.460).

In the next step, we evaluated the associations between the severity of depression symptoms in the final assessment and age, BMI, level of education, week of birth, VO_2_max (at baseline and final), category of PA level (at baseline and final) as well as IPAQ METs (at baseline and final) in both groups. We found no significant correlations in the EDU group. However, in the HIIT group we found significant correlations between the severity of depression symptoms and category of PA level (at baseline) as well as IPAQ METs (at baseline and final). Based on the results of the correlation analysis, we performed series of regression analyses. Details for the significant correlations are presented in the Table 3.

**Table 3:** Predictors of the severity of depression symptoms during second assessment for HIIT group

	Severity of depression symptoms			
	*R*^2^	*F*	*p*-value	*β*
Baseline PA in METs	0.224	9.244	.005	− 0.473
Baseline category of PA	0.219	8.990	.005	− 0.468
Final PA in METs	0.230	9.548	.004	− 0.479

METs—Metabolic equivalents of tasks

PA—Physical activity

### Fear of childbirth

**Fig. 2:**
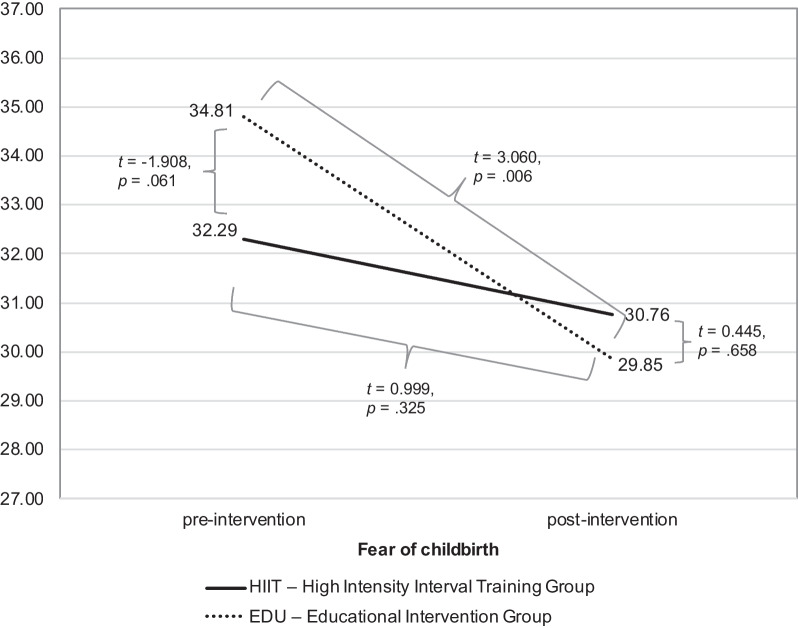
The differences in the severity of fear of childbirth between HIIT and EDU groups pre-and post-intervention the intervention

In case of the fear of childbirth, the ANOVA with repeated measure revealed that there is a significant main effect (*F* = 6.956, *p* = 0.011, η^2^ = 0.118, observed power = 0.735), in the absence of an interaction effect (*F* = 1.710, *p* = 0.197, η^2^ = 0.032, observed power = 0.250); see Fig. 2. Additional, post-hoc analyses pointed that the groups did not differ in the case of the severity of fear of childbirth, both in the first measurement (*t* =  − 1.908, *p* = 0.061, 95% CI: LL =  − 5.160, UL = 0.118, Cohen’s *d* = 0.471; HIIT group: M = 32.29, SD = 5.09; EDU group: M = 34.81 SD = 5.64), as well as in the second measurement (*t* = 0.445, *p* = 0.658, 95% CI: LL =  − 3.211, UL = 5.041, Cohen’s *d* = 0.125; HIIT group: M = 30.76, SD = 7.52; EDU group: M = 29.85, SD = 6.89). The change (decrease in fear of childbirth) between pre- and post-intervention was significant in the EDU group (*t* = 3.060, *p* = 0.006, Cohen’s *d* = 0.684), but not in the HIIT group (*t* = 0.999, *p* = 0.325, Cohen’s *d* = 0.171). However, when controlling for the week of birth (variable entered as a covariate in ANOVA) neither interaction effect (*F* = 2.256, *p* = 0.139, η^2^ = 0.042, observed power = 0.314) nor main effect remained significant (*F* = 1.584, *p* = 0.214, η^2^ = 0.030, observed power = 0.235). In ITT analysis we obtained significant interaction effect (*F* = 4.142, *p* < 0.001, η^2^ = 0.059, observed power = 0.518) and post hoc analyses pointed to the significant between-group difference at baseline (*t* =  − 2.089, *p* = 0.041, 95% CI: LL =  − 5.249, UL =  − 0.119, Cohen’s *d* = 0.507; HIIT group: M = 32.29, SD = 5.09; EDU group: M = 34.97 SD = 5.50).

In the next step, we evaluated the associations between the severity of fear of childbirth in the final assessment and age, BMI, level of education, week of birth, VO_2_max (at baseline and final), category of PA (at baseline and final) as well as Pa in METs (at baseline and final) in both groups. We found no significant correlations in the EDU group. However, in the HIIT group we found significant correlations between the severity of fear of childbirth and category of PA (at baseline) as well as PA in METs (at baseline). Based on the results of the correlation analysis, we performed regression analyses. The details for the significant correlations are presented in Table 4.

**Table 4:** Predictors of the fear of childbirth during final assessment for the HIIT group

	Fear of childbirth			
	*R*^*2*^	*F*	*p*-value	*β*
Baseline PA level in METs	0.145	5.439	.026	− 0.381
Baseline category of PA	0.234	10.294	.003	− 0.493

METs—Metabolic equivalents of tasks

PA—Physical activity

### Physical and mental health

For the assessment of the differences in the physical aspect of health (reflected by the “Physical health” subscale’s score of the SF-12) between groups in the two measurements, repeated ANOVA measures revealed no significant main effect (*F* = 0.015, *p* = 0.903, η^2^ = 0.00, observed power = 0.052) nor interaction (*F* = 0.25, *p* = 0.876, η^2^ = 0.00, observed power = 0.053). Non-significant results were also observed when the week of birth was entered as a covariate in repeated ANOVA measures. Similar outcomes were obtained in the ITT analysis.

**Fig. 3:**
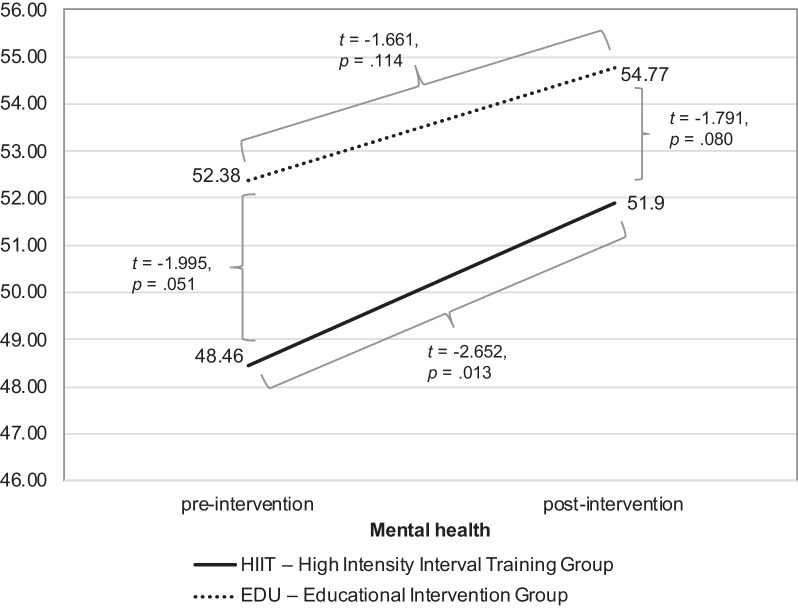
The differences in the mental aspect of health between HIIT and EDU groups pre- and post-intervention

However, in case of the mental aspect of health (reflected by the “Mental health” subscale’s score of the SF-12) the analysis pointed at the significant main effect (*F* = 8.669, *p* = 0.005, η^2^ = 0.153, observed power = 0.823), in the absence of an interaction effect (*F* = 0.099, *p* = 0.755, η^2^ = 0.002, observed power = 0.061) (see Fig. 3). In ITT analysis we also found significant main effect (*F* = 12.163, *p* < 0.001, η^2^ = 0.156, observed power = 0.930), in the absence of an interaction effect (*F* = 0.800, *p* = 0.374, η^2^ = 0.012, observed power = 0.143).

Post-hoc analyses showed that the groups did not differ in the mental aspect of health in the first measurement and in the second measurement (see Table 5 for the details). However, the ITT analysis revealed the significant group difference in the first assessment (*t* =  − 2.098, *p* = 0.40, 95% CI: LL =  − 6.42, UL =  − 0.16, Cohen’s *d* = 0.509).

**Table 5:** Mental health

	Group			95% CI			
	HIIT	EDU	Student’s *t* test	LL	UL	*p*-value	Cohen’s *d*
	n = 34, M ± SD	n = 20, M ± SD					
First measurement	48.46 ± 7.23	52.38 ± 6.53	1.995	− 7.87	− 0.02	0.051	0.562
Second measurement	51.90 ± 6.06	54.77 ± 4.43	− 1.791	− 6.09	0.35	0.080	0.522

Of note, the increase in the mental aspect of health was significant only in the HIIT group (*t* =  − 2.652, *p* = 0.013, 95% CI: LL =  − 5.85, UL =  − 0.76, Cohen’s *d* = 0.476) and non-significant in the EDU group (*t* =  − 1.661, *p* = 0.114, 95% CI: LL =  − 6.04, UL = 0.71, Cohen’s *d* = 0.381). However, when controlling for the week of birth (variable entered as a covariate in ANOVA) neither interaction effect (*F* = 1.236, *p* = 0.272, η^2^ = 0.026, observed power = 0.193) nor main effect remained significant (*F* = 2.271, *p* = 0.106, η^2^ = 0.055, observed power = 0.366). The ITT analysis confirmed the above observations.

In the next step, we evaluated the associations between the physical and mental health in the final assessment and age, BMI, level of education, week of birth, VO_2_max (at baseline and final), category of PA (at baseline and final) as well PA in METs (at baseline and final) in both groups. We found no significant correlations in the control group. However, in the HIIT group we found significant correlations between the physical health and IPAQ (final) as well as between mental health and category of PA (at baseline and final) and PA in METs (at baseline and final). Based on the results of the correlation analysis, we performed regression analyses. The details are presented in Table 6.

**Table 6:** Predictors of the physical and mental health during final assessment for the HIIT group

	*R*^*2*^	*F*	*p*-value	*β*
*Physical health*				
Final PA in METs	0.157	5.947	.020	0.396
*Mental health*				
Baseline category of PA	0.160	5.533	.026	0.400
Baseline PA in METs	0.212	7.799	.009	0.460
Final category of PA	0.127	4.200	.050	0.356
Final PA in METs	0.167	5.818	.022	0.409

PA—Physical activity

METs—Metabolic equivalents of tasks

### Covid-19-related fear

**Fig. 4:**
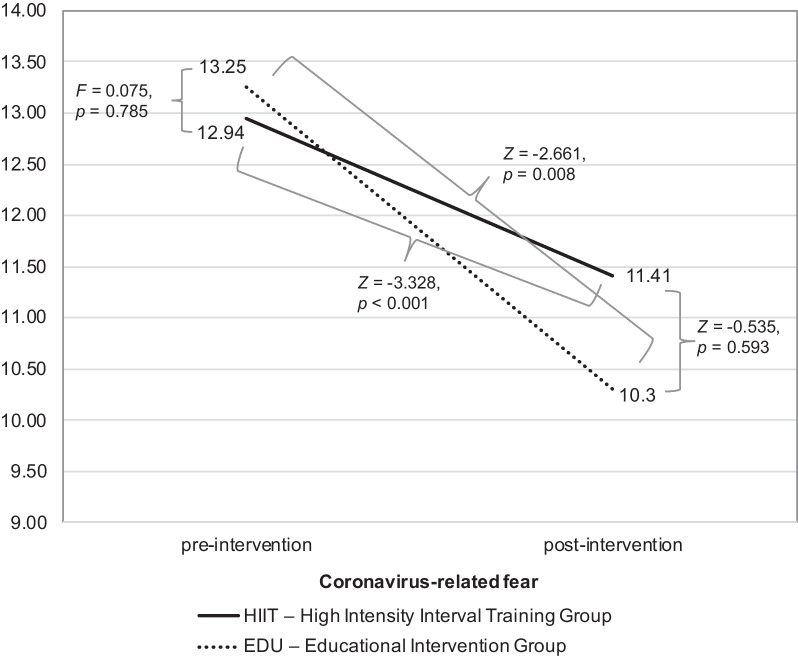
The differences in the severity of the COVID-19-related fear between HIIT and EDU groups pre- and post-intervention

In case of the Covid-19-related fear, the results revealed that there were no significant differences between groups in the first assessment (*F* = 0.075, *p* = 0.785, η^2^ = 0.001, observed power = 0.058; HIIT group: M = 12.94, SD = 0.69; EDU group: M = 13.25, SD = 0.90). Of note, lack of significant results was observed when controlling for the week of birth as well. The analysis with the Mann–Whitney *U* pointed to non-significant results as well (*Z* =  − 0.535, *p* = 0.593; HIIT group: M = 11.41, SD = 0.76; EDU group: M = 10.30, SD = 0.97). The decrease in the fear of Covid-19 (measured with the Wilcoxon *T* test) between the initial assessment and final measurement was significant in the HIIT group (*Z* =  − 3.328, *p* < 0.001) as well as for the EDU group (*Z* =  − 2.661, *p* = 0.008); see Fig. 4. Similar observations were found in the ITT analysis.

In the next step, we evaluated the associations between the COVID-19-related fear in the final assessment and age, BMI, level of education, week of birth, VO_2_max (at baseline and final), category of PA (at baseline and final) as well as PA in METs (at baseline and final) in both groups. We found no significant correlations in the HIIT as well as EDU group.

### The exercise capacity (expressed as VO_2_max)

**Fig. 5:**
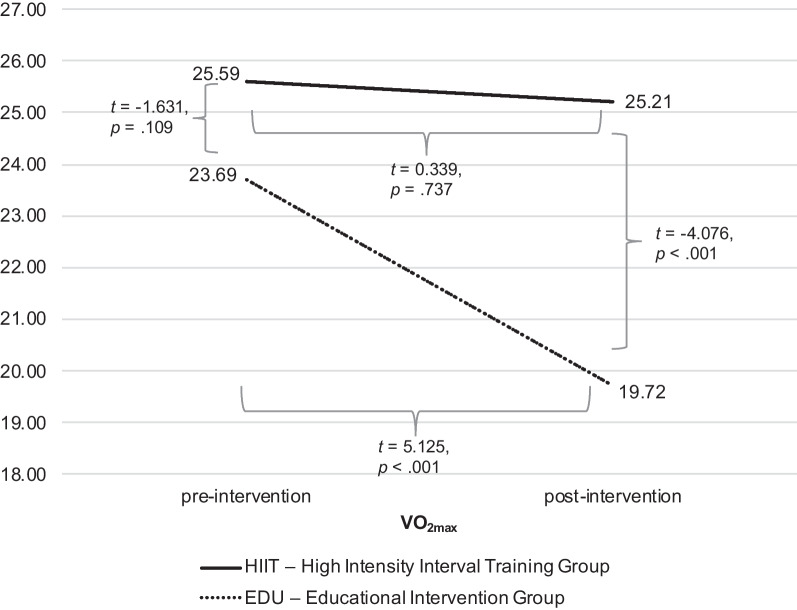
The differences in the VO_2_max between HIIT and EDU groups pre-and post- intervention (in ml/kg/min)

The analysis conducted with repeated measures ANOVA revealed both the significant main effect (*F* = 20.387, *p* < 0.001, η^2^ = 0.290, observed power = 0.993) as well as interaction effect (*F* = 16.928, *p* < 0.001, η^2^ = 0.253, observed power = 0.981), in case of the VO_2_max; see Fig. 5. Post-hoc analyzes showed that the groups (HIIT and EDU) did not differ in the VO_2_max in the first measurement (*t* =  − 1.631, *p* = 0.109, 95% CI: LL =  − 4.241, UL = 0.439, Cohen’s *d* = 0.465; HIIT group: M = 25.59, SD = 4.37; EDU group: M = 23.69, SD = 3.58), however they differed in the second measurement (*t* =  − 4.076, *p* < 0.001, 95% CI: LL =  − 8.191, UL =  − 2.786, Cohen’s *d* = 1.149; HIIT group: M = 25.21, SD = 5.11; EDU group: M = 19.72, SD = 4.14). The change (decrease in VO_2_max) between pre- and post-intervention was significant in the EDU group (*t* = 5.125, *p* < 0.001, 95% CI: LL = 2.348, UL = 5.591, Cohen’s *d* = 1.146), but not in the HIIT group (*t* = 0.339, *p* = 0.737, 95% CI: LL =  − 0.924, UL = 1.292, Cohen’s *d* = 0.060). Similar observations were obtained in the ITT analysis.

### The level of physical activity (based on IPAQ)

Further analysis with Mann–Whitney *U* test revealed that groups did not differ in the IPAQ MET in the first assessment (*Z* = 0.752, *p* = 0.452; HIIT group: M = 2625.27, SD = 1823.21; EDU group: M = 2266.00, SD = 1816.46). Similarly, no between-group differences were observed in the second measurement (*Z* =  − 1.506, *p* = 0.132; HIIT group: M = 3118.31, SD = 1995.15; EDU group: M = 23.69, SD = 3.58). The within-group analyses with the Wilcoxon *T* test revealed, that the differences between initial assessment and second measurement were statistically insignificant for both the HIIT group (*Z* =  − 1.410, *p* = 0.158) and EDU group (*Z* =  − 0.037, *p* = 0.970). Similar observations were obtained in the ITT analysis.

## Discussion

The principal objective of this study was to identify the effects of high-intensity interval training on selected psychological characteristics among pregnant participants. We aimed to estimate the effects of the 8-week supervised online HIIT program on depressive symptoms, fear of childbirth, fear of Covid-19, and perception of quality of life compared to the 8-week educational and self-performed physical activity program. Furthermore, our goal was to determine the predictors of the changes in these parameters.

Before the interpretation of our outcomes it is worth mentioning that participants in both groups were generally in good mental and physical conditions entering the programs, as these were in the inclusion criteria in the project invitation. The study results revealed that the level of depressive symptoms at the beginning of the program was low. Since the participants had satisfactory conditions before starting the program, significantly improving these outcomes was not expected. Nevertheless, we observed a significant reduction of anxiety (fear of childbirth) among EDU group participants. Moreover, an important and novel result is that all participants significantly decreased their level of fear of Covid-19. Neither age, level of education, week of gestation, nor physical activity characteristics were the predictors of those changes. Therefore, we believe that physical activity, regardless of its nature, allowed for reducing negative emotional states among investigated pregnant individuals. We also assume that daily adaptation to the pandemic situation could influence the increase of emotional resilience among studied participants. Nonetheless, researchers warn that pregnant women, for whom coronavirus had a more significant psychological impact, are more likely to suffer from anxiety and depressive symptoms also associated with reduced attachment to the unborn baby [61]. Therefore, all the interventions that help lower Covid-19 -related fear could work as a protective factor against affective problems.

Our study is in line with the works by other authors who attempted to find solutions for the maintenance of proper health status of future mothers during the current global situation. For example, Hillyard et al. [62] in the studies on 553 pregnant women with gestational diabetes, underlined the urgent need for targeted public health initiatives to increase physical activity and reduce sedentary behaviors as the pandemic continues and for future lockdowns. Pregnant women should be encouraged to participate in online classes, prepared by qualified exercise professionals and coaches, which could be beneficial in lowering Covid-19 anxiety by reducing face-to-face meetings [62, 63]. Our outcomes confirm the effectiveness of online exercise offer in this regard.

However, the current study's findings revealed no significant intergroup differences in depression symptoms in the first and second assessments and no changes over time. As long as participants were non-clinical samples, the depressive symptoms were negligible and therefore hard to change. Nonetheless, the authors observed a substantial improvement in mental health among HIIT group. The current literature underlines that mental health is a strong predictor of anxiety, especially in pregnant women who are vulnerable to anxiety disorders compared to non-pregnant women. Consequently, all the interventions which are proven to boost mental health seem to be very valuable [64, 65]. Those results are remarkable findings considering previous studies that pregnancy has been established as period of vulnerability for psychological and social status changes, which increase the potential risk of impaired physical and mental health [1]. Furthermore, the severity of depression symptoms in the HIIT group was associated with a category of physical fitness and physical activity levels pre- and post-intervention. Our findings correspond to the observations of other authors that regular physical activity leads to the reduction of depressive and anxiety disorders in women in the perinatal period, especially among women experiencing mild or moderate depressive conditions [66, 67]. While interpreting our results, one can refer to other authors who observed that moderate-intensity programs were more effective in treating depression and reducing anxiety than high-intensity exercise. Following this interpretation, moderate intensity continuous training programs would be a better recommendation for women with depression. Nonetheless, Ong et al. [68], while investigating the influence of continuous cycling exercise at a steady power output compared to interval cycling consisting of continuous cycling at the same power output but with the addition of six 15-s self-paced higher intensity efforts showed that interval cycling significantly increased enjoyment among pregnant women at late pregnancy [68].

Our findings highlight that both interventions seem to be an efficient way to preserve the quality of life along the course of pregnancy. Despite not only the lower level of quality of life reported by pregnant women when compared with non-pregnant women of the same age [4] and the decrease of the physical aspect of quality of life along the course of pregnancy [5], both the HIIT program and the educational program could be considered in the antenatal care, at least to maintain quality of life perceived by women throughout pregnancy. The improvements obtained in the mental aspect of quality of life by the HIIT program group, which is consistent with meeting physical activity guidelines [69], can be explained not only by its stable behavior during pregnancy [5]. It is also related to the role of exercise practice on the promotion of social interactions [1] and also by the positive influence of exercise on several mental disorders like depression or anxiety [7].

Another important benefit of our HIIT intervention is that the HIIT program compared to EDU program allowed to maintain cardiorespiratory fitness levels (CRF). The meta-analysis of Stubbs et al. [70], proved that people with depression, including major depressive disorder, could increase CRF in response to exercise interventions. This outcome may be very important for the pregnant population, where the prevalence of depression is very high [11]. In our study the HIIT group maintained the VO_2_max level despite the progression of pregnancy and in contradiction to the EDU group which presented significantly worse VO_2_max values after 8 weeks. The EDU group responded in line with the works by others authors. They explained that the cardiorespiratory fitness decline as pregnancy advances is a typical ventilatory response related to the elevated metabolic costs of exercise with the pregnant body [71]. Taking into account our data, HIIT programs should be recommended for pregnant women to maintain their cardiorespiratory fitness level throughout pregnancy. This may support the proper supply of oxygen to the fetus and, consequently, its development [45]. However, based on the outcomes of our study, we should limit our recommendations to pregnant individuals in good physical and mental health. Moreover, the HIIT program could be more enjoyable for women who prefer to be physically active (with supervision by an exercise professional) than the EDU program. Many participants in the EDU group resigned from completing the program, probably because the intervention was educational only.

There are limitations of the study. The main limitation concerns the aspects related to the testing of physical activity levels in the study participants. Self-reported assessment of PA may be subject to social desirability bias; however, PA was collected using a validated questionnaire, which minimized potential bias. Moreover, following the observations of Domingues et al. [72], the results might have been influenced (even unconsciously) by aspects of social pressure and women’s fear that they exercise too hard and that it could lead to a miscarriage or harm the baby. Hence, it could reduce the therapeutic effectiveness of exercises in reducing depression-anxiety states through physical activity. Additionally, it is unclear which part of the programs (exercise or education) was more effective in decreasing fear of Covid-19, as both groups recorded a significantly favorable result. Another limitation is the aspect related to the study group itself. Some women missed the second assessment. Thus, missing data led to a lower sample size and therefore reduced power to detect differences in change between groups. The educational and social characteristics of both groups were pretty similar, not allowing the extrapolation of data for other groups. Finally, the fear of COVID-19 questionnaire is very recent and was neither previously validated for pregnant women nor do we have reference data yet. Future experiments on HIIT program efficiency should consider different study groups with more diverse mental health and psychological characteristics. One group could be pregnant participants without affective problems, where HIIT program would be a form of prevention from emotional difficulties during pregnancy. The comparative group could be the future mothers with higher levels of anxiety and depression as a form of intervention to reduce mental problems.

## Conclusions

The study revealed that all the outcomes progress along with the interventions. Very positive trends in the decrease in the severity of depressive symptoms, fear of childbirth, fear of Covid-19, and the increase of physical activity level and mental health among future mothers were observed in the current study, not to mention the significant increase in cardiovascular fitness levels of the HIIT group. High intensity interval training may be beneficial for healthy women as a form of physical activity that is safe and beneficial for an uncomplicated pregnancy and has a preventive effect on depression and anxiety symptoms. However, more research is needed to determine the effectiveness of prenatal HIIT in pregnant women in various psychological conditions.

## Data Availability

We performed no data manipulations. Materials for this study are available by emailing the corresponding author (DW) or head of the project (AS). The data analysis presented in this work was not preregistered.

## Abbreviations

BMI: Body mass index
EDU: Educational program
HIIT: High intensity interval training
MET: Metabolic equivalent of task
SF-12: 12-Item short form health survey
IPAQ: International physical activity questionnaire
VO_2_max: Maximal oxygen uptake

## References

[1] Marcus SM (2009). Depression during pregnancy: rates, risks and consequences–Motherisk update. Can. J. Clin. J. Can. Pharmacol. Clin..

[2] Saisto T, Halmesmäki E (2003). Fear of childbirth: a neglected dilemma. Acta Obstet Gynecol Scand.

[3] Oviedo-Caro MA, Bueno-Antequera J, Munguía-Izquierdo D (2021). The associations of pregnancy-related symptoms with health-related quality of life at midpregnancy: the PregnActive project. J. Matern. Fetal Neonatal Med..

[4] Chang S-R (2014). A repeated measures study of changes in health-related quality of life during pregnancy and the relationship with obstetric factors. J Adv Nurs.

[5] Lagadec N (2018). Factors influencing the quality of life of pregnant women: a systematic review. BMC Pregnancy Childbirth.

[6] Davenport MH (2019). Impact of prenatal exercise on maternal harms, labour and delivery outcomes: a systematic review and meta-analysis. Br J Sports Med.

[7] Davenport MH (2018). Impact of prenatal exercise on both prenatal and postnatal anxiety and depressive symptoms: a systematic review and meta-analysis. Br J Sports Med.

[8] Davenport MH (2018). Impact of prenatal exercise on neonatal and childhood outcomes: a systematic review and meta-analysis. Br J Sports Med.

[9] Davenport MH (2018). Prenatal exercise for the prevention of gestational diabetes mellitus and hypertensive disorders of pregnancy: a systematic review and meta-analysis. Br J Sports Med.

[10] Okafor UB, Goon DT (2020). Physical activity and exercise during pregnancy in Africa: a review of the literature. BMC Pregnancy Childbirth.

[11] Haßdenteufel K (2020). Reduction in physical activity significantly increases depression and anxiety in the perinatal period: a longitudinal study based on a self-report digital assessment tool. Arch Gynecol Obstet.

[12] Murray L (2011). Maternal postnatal depression and the development of depression in offspring up to 16 years of age. J Am Acad Child Adolesc Psychiatry.

[13] Kołomańska D, Zarawski M, Mazur-Bialy A (2019). Physical activity and depressive disorders in pregnant women—A systematic review. Medicina (Mex.).

[14] Vargas-Terrones M, Barakat R, Santacruz B, Fernandez-Buhigas I, Mottola MF (2019). Physical exercise programme during pregnancy decreases perinatal depression risk: a randomised controlled trial. Br J Sports Med.

[15] Guszkowska M (2014). The effect of exercise and childbirth classes on fear of childbirth and locus of labor pain control. Anxiety Stress Coping.

[16] Guszkowska M, Langwald M, Zaremba A, Dudziak D (2014). The correlates of mental health of well-educated Polish women in the first pregnancy. J Ment Health Abingt Engl.

[17] Krzepota J, Sadowska D, Biernat E (2018). Relationships between physical activity and quality of life in pregnant women in the second and third trimester. Int J Environ Res Public Health.

[18] Oviedo-Caro MA, Bueno-Antequera J, Munguía-Izquierdo D (2018). Explanatory factors and levels of health-related quality of life among healthy pregnant women at midpregnancy: a cross-sectional study of The PregnActive Project. J Adv Nurs.

[19] Davenport MH, Meyer S, Meah VL, Strynadka MC, Khurana R (2020). Moms are not OK: COVID-19 and maternal mental health. Front Glob Womens Health.

[20] Gildner TE, Laugier EJ, Thayer ZM (2020). Exercise routine change is associated with prenatal depression scores during the COVID-19 pandemic among pregnant women across the United States. PLoS ONE.

[21] Atkinson L (2020). Encouraging physical activity during and after pregnancy in the COVID-19 era, and beyond. Int J Environ Res Public Health.

[22] Ding W (2021). Knowledge, attitudes, practices, and influencing factors of anxiety among pregnant women in Wuhan during the outbreak of COVID-19: a cross-sectional study. BMC Pregnancy Childbirth.

[23] Kahyaoglu Sut H, Kucukkaya B (2021). Anxiety, depression, and related factors in pregnant women during the COVID-19 pandemic in Turkey: a web-based cross-sectional study. Perspect Psychiatr Care.

[24] Mappa I, Distefano FA, Rizzo G (2020). Effects of coronavirus 19 pandemic on maternal anxiety during pregnancy: a prospectic observational study. J Perinat Med.

[25] Verweij EJ, Mhamdi HI, Steegers EAP, Reiss IKM, Schoenmakers S (2020). Collateral damage of the covid-19 pandemic: a Dutch perinatal perspective. BMJ.

[26] Saccone G (2020). Psychological impact of coronavirus disease 2019 in pregnant women. Am J Obstet Gynecol.

[27] Lebel C, MacKinnon A, Bagshawe M, Tomfohr-Madsen L, Giesbrecht G (2020). Elevated depression and anxiety symptoms among pregnant individuals during the COVID-19 pandemic. J Affect Disord.

[28] Zilver SJM (2021). Stress, anxiety and depression in 1466 pregnant women during and before the COVID-19 pandemic: a Dutch cohort study. J Psychosom Obstet Gynecol.

[29] ACOG Committee Opinion Summary (2020). Number 804: Physical activity and exercise during pregnancy and the postpartum period. Obstet Gynecol.

[30] ACSM, ACSM information on pregnancy physical activity. In: American College of Sports Medicine; 2020.

[31] Mottola M (2018). 2019 Canadian guideline for physical activity throughout pregnancy. Br J Sports Med.

[32] WHO. WHO guidelines on physical activity and sedentary behaviour. In (Vol. 57). Geneva: World Health Organization; 2020

[33] Borg G. Borg’s perceived exertion and pain scales. viii, 104. Human Kinetics; 1998.

[34] Davenport MH (2020). Exercise during pregnancy: a prescription for improved maternal/fetal well-being. ACSMs Health Fit J.

[35] Rodriguez-Ayllon M (2021). Associations of physical activity, sedentary time, and physical fitness with mental health during pregnancy: the GESTAFIT project. J Sport Health Sci.

[36] Thompson WR (2022). Worldwide survey of fitness trends for 2022. ACSMs Health Fit J.

[37] Wood G, Murrell A, van der Touw T, Smart N (2019). HIIT is not superior to MICT in altering blood lipids: a systematic review and meta-analysis. BMJ Open Sport Exerc Med.

[38] Heggelund J, Kleppe KD, Morken G, Vedul-Kjelsås E (2014). High aerobic intensity training and psychological states in patients with depression or schizophrenia. Front Psychiatry.

[39] Borrega-Mouquinho Y, Sánchez-Gómez J, Fuentes-García JP, Collado-Mateo D, Villafaina S (2021). Effects of high-intensity interval training and moderate-intensity training on stress, depression, anxiety, and resilience in healthy adults during coronavirus disease 2019 confinement: a randomized controlled trial. Front Psychol.

[40] Plag J, Ergec D-L, Fydrich T, Ströhle A (2019). High-intensity interval training in panic disorder patients: a pilot study. J Nerv Ment Dis.

[41] Paolucci EM, Loukov D, Bowdish DME, Heisz JJ (2018). Exercise reduces depression and inflammation but intensity matters. Biol Psychol.

[42] Kleinert J, Bassek M (2019). Psychological aspects of high intensity interval training (HIIT) in therapy: a literature review. [in German] Psychologische Aspekte von Hochintensivem Intervalltraining (HIIT) in der Therapie: Eine Übersicht der Literatur. BG Bewegungstherapie Gesundheitssport.

[43] Szumilewicz A (2022). How to HIIT while pregnant? The protocol characteristics and effects of high intensity interval training implemented during pregnancy—a systematic review. Balt J Health Phys Act.

[44] Skow R (2018). Effects of prenatal exercise on fetal heart rate, umbilical and uterine blood flow: A systematic review and meta-analysis. Br J Sports Med.

[45] Melzer K, Schutz Y, Boulvain M, Kayser B (2010). Physical activity and pregnancy: cardiovascular adaptations, recommendations and pregnancy outcomes. Sports Med Auckl NZ.

[46] Beck AT, Steer RA, Brown GK. BDI-II, Beck depression inventory: manual. Harcourt Brace; 1996.

[47] Areskog B, Kjessler B, Uddenberg N (1982). Identification of women with significant fear of childbirth during late pregnancy. Gynecol Obstet Invest.

[48] Ware J, Kosinski M, Keller SD (1996). A 12-item short-form health survey: construction of scales and preliminary tests of reliability and validity. Med Care.

[49] Ahorsu DK (2020). The fear of COVID-19 scale: development and initial validation. Int J Ment Health Addict.

[50] Craig CL (2003). International physical activity questionnaire: 12-country reliability and validity. Med Sci Sports Exerc.

[51] Cheng HL (Helen) A simple, easy-to-use spreadsheet for automatic scoring of the International Physical Activity Questionnaire (IPAQ) Short Form; 2016. 10.13140/RG.2.2.21067.80165

[52] IPAQ. Guidelines for data processing and analysis of the international physical activity questionnaire (ipaq)-short and long forms. Int Phys Act Quest (2005)

[53] Szumilewicz A (2019). Acute postexercise change in circulating irisin is related to more favorable lipid profile in pregnant women attending a structured exercise program and to less favorable lipid profile in controls: an experimental study with two groups. Int J Endocrinol.

[54] Beaver WL, Wasserman K, Whipp BJ (1986). A new method for detecting anaerobic threshold by gas exchange. J Appl Physiol.

[55] Persinger R, Foster C, Gibson M, Fater DCW, Porcari JP (2004). Consistency of the talk test for exercise prescription. Med Sci Sports Exerc.

[56] Santos-Rocha R, Gutiérrez IC, Szumilewicz A, Pajaujiene S, Santos-Rocha R (2019). Exercise testing and prescription for pregnant women. Exercise and sporting activity during pregnancy: evidence-based guidelines.

[57] Szumilewicz A, Santos-Rocha R, Santos-Rocha R (2019). Exercise selection and adaptations during pregnancy. Exercise and sporting activity during pregnancy: evidence-based guidelines.

[58] Santos-Rocha R, Szumilewicz A, Perales M, Pajaujiene S, EuropeActive standards EQF level 5—pregnancy and postnatal exercise specialist. EuropeActive (2016).

[59] Nosek BA (2015). Promoting an open research culture. Science.

[60] Moher D (2012). CONSORT 2010 explanation and elaboration: updated guidelines for reporting parallel group randomised trials. Int J Surg Lond Engl.

[61] Filippetti ML, Clarke ADF, Rigato S (2022). The mental health crisis of expectant women in the UK: effects of the COVID-19 pandemic on prenatal mental health, antenatal attachment and social support. BMC Pregnancy Childbirth.

[62] Hillyard M, Sinclair M, Murphy M, Casson K, Mulligan C (2021). The impact of COVID-19 on the physical activity and sedentary behaviour levels of pregnant women with gestational diabetes. PLOS ONE.

[63] Rabiepoor S, Rezavand S, Yas A, Ghanizadeh N (2019). Influential factors in physical activity amongst pregnant women. Balt J Health Phys Act.

[64] Uguz F, Yakut E, Aydogan S, Bayman MG, Gezginc K (2019). Prevalence of mood and anxiety disorders during pregnancy: a case-control study with a large sample size. Psychiatry Res.

[65] Yang J, Qu J, Sun K, Gao L-L (2021). Anxiety symptoms and health-related quality of life in mainland Chinese pregnant women: a cross-sectional study. J. Reprod. Infant Psychol.

[66] Perales M, Refoyo I, Coteron J, Bacchi M, Barakat R (2015). Exercise during pregnancy attenuates prenatal depression: a randomized controlled trial. Eval Health Prof.

[67] Teychenne M, York R (2013). Physical activity, sedentary behavior, and postnatal depressive symptoms: a review. Am J Prev Med.

[68] Ong MJ, Wallman KE, Fournier PA, Newnham JP, Guelfi KJ (2016). Enhancing energy expenditure and enjoyment of exercise during pregnancy through the addition of brief higher intensity intervals to traditional continuous moderate intensity cycling. BMC Pregnancy Childbirth.

[69] Oviedo-Caro MA, Bueno-Antequera J, Munguía-Izquierdo D (2022). Meeting physical activity guidelines and its association with health-related quality of life throughout pregnancy: the PregnActive project. Psychol Health Med.

[70] Stubbs B, Rosenbaum S, Vancampfort D, Ward PB, Schuch FB (2016). Exercise improves cardiorespiratory fitness in people with depression: a meta-analysis of randomized control trials. J Affect Disord.

[71] Davenport MH, Steinback CD, Mottola MF (2009). Impact of pregnancy and obesity on cardiorespiratory responses during weight-bearing exercise. Respir Physiol Neurobiol.

[72] Domingues MR, Matijasevich A, Barros AJD (2009). Physical activity and preterm birth: a literature review. Sports Med.

